# Genotype imputation methods for whole and complex genomic regions utilizing deep learning technology

**DOI:** 10.1038/s10038-023-01213-6

**Published:** 2024-01-15

**Authors:** Tatsuhiko Naito, Yukinori Okada

**Affiliations:** 1 Department of Statistical Genetics, Osaka University Graduate School of Medicine, 2-2, Yamadaoka, Suita-shi, Osaka 565-0871 Japan; 2 Laboratory for Systems Genetics, RIKEN Center for Integrative Medical Sciences, 1-7-22, Suehiro-cho, Tsurumi-ku, Yokohama City, Kanagawa 230-0045 Japan; 3 Department of Genome Informatics, Graduate School of Medicine, the University of Tokyo, 7-3-1, Hongo, Bunkyo-ku, Tokyo, 113-8655 Japan; 4 Integrated Frontier Research for Medical Science Division, Institute for Open and Transdisciplinary Research Initiatives, Osaka University, 2-2, Yamadaoka, Suita-shi, Osaka 565-0871 Japan; 5 Premium Research Institute for Human Metaverse Medicine (WPI-PRIMe), Osaka University, 2-2, Yamadaoka, Suita-shi, Osaka 565-0871 Japan

**Keywords:** Genome-wide association studies, Genome informatics

## Abstract

The imputation of unmeasured genotypes is essential in human genetic research, particularly in enhancing the power of genome-wide association studies and conducting subsequent fine-mapping. Recently, several deep learning-based genotype imputation methods for genome-wide variants with the capability of learning complex linkage disequilibrium patterns have been developed. Additionally, deep learning-based imputation has been applied to a distinct genomic region known as the major histocompatibility complex, referred to as HLA imputation. Despite their various advantages, the current deep learning-based genotype imputation methods do have certain limitations and have not yet become standard. These limitations include the modest accuracy improvement over statistical and conventional machine learning-based methods. However, their benefits include other aspects, such as their “reference-free” nature, which ensures complete privacy protection, and their higher computational efficiency. Furthermore, the continuing evolution of deep learning technologies is expected to contribute to further improvements in prediction accuracy and usability in the future.

## Introduction

The research investigating the impact of genetic variations on complex human traits has witnessed remarkable progress in recent years, which can largely be attributed to the advent of genome-wide association studies (GWAS). GWAS enables the identification of associations of genotypes with target phenotypes by testing for differences in the allele frequency of genome-wide genetic variants between phenotypically different individuals [1]. This has been facilitated by genotyping arrays that can simultaneously collect genotype data covering tens of thousands to millions of single-nucleotide polymorphisms (SNPs) within individual samples at relatively low costs. However, a single chip possesses the ability to collect genotypes for a smaller percentage of whole-genome variants [2]. Hence, achieving wider coverage of variants is warranted for not missing significant associations and to enhance the power of GWAS, and also for identifying causal variants directly associated with the phenotypes of interest (i.e., fine-mapping) [3, 4]. While whole-genome sequencing is optimal for these purposes, it remains expensive and presents technical challenges for very large sample sizes. Therefore, genotypes for unmeasured variants are generally inferred using inter-variant correlations (i.e., linkage disequilibrium, LD) constructed from reference panels to facilitate the maximal coverage of variants. This procedure known as genotype imputation, also enables the integration of different genotyping platforms, allowing exploration of previously unattainable sample sizes.

Majority of the current standard genotype imputation tools use statistical or conventional machine learning methods to infer genotypes of each variant based on predefined haplotype hypotheses [5, 6]. Deep learning techniques have recently emerged as a powerful paradigm in various research and industrial domains [7]. The deep learning models are able to extract intricate patterns and learn complex intervariable relationships from vast amounts of data, and as a result have achieved a higher prediction accuracy in a wide variety of fields when compared to statistical and conventional machine learning methods. Indeed, deep learning has been applied to develop novel genotype imputation methods based on of the assumption that these models could learn complex LD patterns. In addition, deep learning-based imputation has been further applied to the major histocompatibility complex (MHC), which is a distinct genomic region, specifically referred to as human leukocyte antigen (HLA) imputation. After introducing basic knowledge about genotypic imputation, this review describes the currently available deep learning-based genotype and HLA imputation methods, focusing on their specific adaptations for imputation tasks, as well as the underlying deep learning models. Moreover, this review also addresses the challenges, advantages, and future directions regarding deep learning-based genotype imputation.

## Genotype imputation in human genetic studies

Genotype imputation infers genotypes at ungenotyped, mainly single nucleotide variants and short indels, or missing genotypes in target sample sets using LD structure from phased haplotype reference panels comprising samples with denser genetic maps, typically from whole-genome sequencing. Current standard genotype imputation tools, including the Impute5 [8], Minimac4 [9], and Beagle5.4 [10], employ the Li and Stephens haplotype model [11] to infer genotypes of each variant. This model proposes that the genome sequence of an individual can be represented by recombination and a small number of mutations from those of other individuals. Thus, these tools estimate haplotypes that match the input genotypes by considering the recombination of haplotypes present in the reference panels to infer genotypes of unobserved variants. Hidden Markov models (HMMs) are practically used to impute, where the observed processes of the HMMs are represented by the observed genotypes of unknown phase in a study sample, while the hidden states of the HMMs are represented by an underlying and unobserved set of phased genotypes. Independent benchmarking reported the competitive imputation accuracy of these tools, with a sensitivity of over 97 and 99% for minor allele frequency of greater and less than 5% respectively [12]. Specifically, common variants were more accurately detected by Beagle 5.4, while low frequency and rare variants were better imputed by Impute5 and Minimac4. As for computational burden, the shortest processing time was demonstrated by Beagle5.4 when compared to Minimac4 and Imput5, while the least memory was utilized by Minimac4. Minimac4 and Impute5 can take advantage of a two-step process for alleviating computational burden: target sample genotypes are haplotype phased before imputation, which is referred to as pre-phasing. The history, methodologies, and applications of these conventional genotype imputation methods have been intensively reviewed in some previous literature [5, 6].

Reference panels have also been updated along with the methodological development. The HapMap Consortium [13] and 1000 Genomes Project (1KGP) [14] are the widely used sources of haplotype reference panels in GWASs. More than 80 million variants on all autosomes and the X chromosome from 2504 individuals comprising of 26 different ancestry populations can be found in the phase III 1KGP reference panel. It is necessary to employ reference panels consisting of individuals from the same ancestral groups as the target samples due to the inter-ancestry variations in haplotype structures. Thus, multi-ancestral resources have been crucial in facilitating accurate imputation and enabling subsequent studies for diverse ancestral populations. The Haplotype Reference Consortium (HRC), which offers 40 million variants from more than 30 thousand samples has practically replaced these resources [15]. The HRC reference panels improve imputation for samples with undetected admixture ancestry or rare haplotypes by incorporating data from the multi-ancestry 1KGP individuals.

Genotype imputation servers enable users to perform genotype imputation remotely solely by uploading genotype data of target samples. These servers eliminate the need to obtain reference panels and computational skills required to implement imputation pipelines, thereby streamlining human genetic research. The Michigan Imputation Server, provided by the University of Michigan, is a secure cloud-based imputation platform that incorporates Minimac3 for imputation [9], while the Wellcome Sanger Institute provides a comparable imputation platform, the Sanger Imputation Service [15], employing PBWT for imputation [16]. Both servers provide various reference panels, including the 1KGP and HRC.

## Deep learning-based genotype imputation methods

Deep learning is a type of machine learning that simulates the way the human brain processes information to perform tasks by using artificial neural networks [7]. These neural networks consisting of multiple interconnected layers, which are basically input, hidden, and output layers, enable extraction and learning of complex features from data. The two main steps involved in the application of deep learning models are: 1) training models with input and correctly labeled output data, and 2) using the trained models to predict outputs based on target input data. Thus, in a general workflow involved in the application of deep learning to genotype imputation, reference panels are used to train models, with limited genotypes as inputs for predicting target genotypes as outputs. The trained models are then applied to impute the genotype data of target individuals. Most of the current deep learning-based methods output the target genotype corresponding to a particular input haplotype. pre-phasing of the input genotype data by other software is necessary, potentially impacting imputation accuracy. Existing deep learning-based imputation methods have been summarized in Table 1.

**Table 1:** Existing deep learning-based methods for genotype and HLA imputation

Reference	Name	Target	Methods	URL
Chen et al. [17]	SDCA	Genotype imputation	Autoencoder	https://github.com/work-hard-play-harder/SCDA
Song et al. [18]	(Unnamed)	Genotype imputation	Autoencoder	https://github.com/mengsong28/Autoencoder_imputation
Dias et al. [19]	(Unnamed)	Genotype imputation	Autoencoder	https://github.com/TorkamaniLab/Imputation_Autoencoder
Kojima et al. [20]	RNN-IMP	Genotype imputation	Recurrent neural network	https://github.com/kanamekojima/rnnimp
Mowlaei et al. [25]	STI	Genotype imputation	Self-attention neural networks	https://github.com/shilab/STI
Naito et al. [34]	Deep*HLA	HLA imputation	Convolutional neural networks	https://github.com/tatsuhikonaito/DEEP-HLA
Tanaka et al. [38]	HLARIMNT	HLA imputation	Self-attention neural networks	https://github.com/seitalab/HLARIMNT

**Fig. 1:**
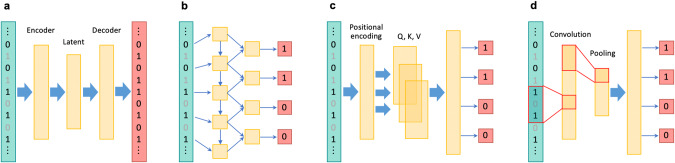
Simplified application of different deep learning architectures to genotype imputation. In each illustration, the leftmost layer represents the input; the genotypes of input variants have been shown in black and the genotypes of target missing variants are gray. The middle and rightmost layers depict the hidden and output layers, respectively. **a** An autoencoders-based approach: An encoder compresses input data into a lower-dimensional representation and a decoder reconstructs the original data from the compressed representation. Genotypes of target variants are masked (collapsed) in genotype imputation. **b** An RNNs-based approach: Each variant is sequentially processed to predict genotypes of next variants by the self-loop structures. To note, the figure displays a unidirectional RNN model, whereas RNN-IMP employs bidirectional RNNs. **c** A self-attention-based approach: Input data undergo positional embedding and are transformed into three types of vectors: query, key, and value. The normalized similarity scores between the query and key vectors are then applied to the value vectors of other data points, resulting in a weighted sum, which represents the contextualized representation of the current data point. **d** A CNNs-based approach: Convolutional filters capture local features and pooling layers down-sample features. Fully-connected layers are typically used to integrate pre-step layers to make final predictions

An autoencoder, which is a type of a neural network used for unsupervised learning, that aims to learn a compressed representation of the input data, was used in the first attempt to apply deep learning models to genotype imputation [17]. An autoencoder consists of an encoder, which performs the function of compressing the data into a lower-dimensional representation, and a decoder, which reconstructs the original data from the compressed representation (Fig. 1a). Autoencoders are commonly used for dimensionality reduction, feature learning, data denoising, and anomaly detection. Chen et al. proposed a sparse convolutional denoising autoencoder (SDCA), which analyzes corrupted input data with missing genotypes and reconstructs the output. The encoder of the SDCA extracts essential features and learn a robust representation of the LD structure via convolutional kernels [17]. Convolution in deep learning is described in the subsequent section. Song et al. implemented a customized training loop with modification of the training process involving only a single batch loss, thereby resulting in a superior imputation accuracy over SDCA [18]. The autoencoder-based approach developed by Dias *et al*. employed a large, commonly used reference panel that spanned an entire human chromosome [19]. Their method achieved superior imputation accuracy compared to the standard imputation tools across different allele frequency spectra and ancestries. Notably, their unique encoding method uses unphased genotype data as input, eliminating the dependency on pre-phasing performed by other tools, unlike the other methods.

RNN-IMP is a genotype imputation method that employs recurrent neural networks (RNNs) [20]. RNNs are designed to process sequential data by maintaining hidden states that capture temporal information (Fig. 1b). Due to the presence of loops in their architecture, RNNs can persistently process and store information from previous time steps, unlike traditional feedforward neural networks. Natural language processing is a typical application of RNNs. Application of RNNs to genotype imputation is reasonable, given the sequential nature of genotype data. RNN-IMP employs a bidirectional longer short-term memory [21] with gated recurrent unit [22] in its architecture. RNN-IMP was competitive with the standard tools which explicitly require reference panels and outperformed them when imputing genotypes of East Asian individuals on the condition that East Asian individuals were excluded from the 1KGP reference panel to simulate de-identification.

Attention is a mechanism that enables a model to focus on specific parts of input data that are the most relevant for the task [23], and enhances the model’s ability to selectively process information by assigning different weights or importance to different elements of the input. Transformer is an attention-based deep learning architecture, which relies solely on self-attention mechanisms, enabling the capturing of long-range dependencies in the input sequence (Fig. 1c). This architecture has demonstrated a stellar performance across various tasks, primarily natural language processing, thereby enhancing the importance of attention mechanisms [24]. Mowlaei et al. recently developed STI, a Transformer-based genotype imputation method, which demonstrated significantly higher imputation accuracy compared to the current standard software and other deep learning-based methods [25].

## Application of deep learning to imputation in distinct genomic regions represented by the MHC

Certain genomic regions have unique LD structures and variant compositions, exemplified by the MHC region, necessitating genotype imputation methods tailored to their unique characteristics. The MHC region is located at 6p21.3 and encodes multiple genes related to immune responses and inflammatory pathways [26, 27]. The highest number of disease associations in GWAS, particularly autoimmune diseases, have been reported for the MHC region [28]. Among the genes in the MHC region, HLA genes, which are involved in the key role of presenting antigens to T cells, have been considered to explain most risk (i.e., heritability) of the MHC. Particularly, the risk in HLA variants is presumed to be attributed mainly to amino acid variants of HLA proteins or their combinations (i.e,. 4-digit HLA alleles) since they can directly affect the antigen binding and recognition. Therefore, comprehensive HLA allelic typing for target individuals is needed to perform fine-mapping this region. However, genotyping the MHC region requires specialized techniques due to its high degree of polymorphism and structural variants [29], and these techniques are often expensive. Thus, imputation-based approaches known as HLA imputation are often resorted to for determining genotypes and HLA allelic types based on LD between such HLA variants and regional SNPs. HLA imputation methods have conventionally followed the same principle as general genotype imputation [30], occasionally incorporating the standard genotype imputation software optimized for HLA imputation [31]. Existing HLA imputation methods and insights obtained from fine-mapping the MHC region have been described elsewhere [32]. The overall concordance rate between imputed and correct alleles for each HLA gene is generally greater than 90% for widely-used software, when high-quality reference panels are used [33]. However, the accuracy tends to be lower for less frequent alleles or hyper-multi-allelic genes, such as *HLA-B* and *HLA-DRB1*.

Deep*HLA is an HLA imputation tool that employs convolutional neural networks (CNNs) in its architecture [34]. CNNs consist of two key components: 1) convolutional layers that take the input data with small filters for detecting patterns or local features and 2) pooling layers that down-sample feature maps by summarizing the presence of features (Fig. 1d). With notable achievements in image recognition, CNNs have been applied to diverse domains [35]. The rationale behind the development of Deep*HLA was that the ability of CNNs to learn local complex features would enable capturing of the intricate LD structure of the MHC region. Its architecture employs multi-task learning, which simultaneously imputes alleles of multiple HLA genes belonging to the same preset LD-based groups. Deep*HLA outperformed other methods, particularly for imputing low-frequency and rare alleles. The abundance of rare alleles in the HLA genes makes this a rather valuable advantage, and is all the more beneficial when conducting cross-ancestry fine-mapping, as different ancestry datasets need to be integrated despite diverse allele frequency spectra between ancestries. Application of Deep*HLA to the cross-ancestry fine-mapping of type 1 diabetes revealed risk-associated HLA variants shared across different ancestries. Deep*HLA was found to be computationally efficient enough to be applied to biobank-scale data. Deep*HLA has already been practically applied for fine-mapping in the MHC region for different diseases [36, 37]. In the more recent years, Transformer has been deployed also in HLA imputation as HLARIMNT, has achieved higher imputation accuracy than Deep*HLA [38]. This suggests that self-attention can successfully be applied to capture the long-range LD structure of the MHC region, and make finer predictions.

## Challenges, advantages, and future perspectives for deep learning-based genotype imputation

Despite their proposed advantages, deep learning-based imputation methods have not yet been accepted as standard. One plausible explanation for this can be attributed to the fact that current deep learning-based methods demonstrated only modest improvements in prediction accuracy over widely used conventional methods. This contradicts the observations that deep learning models outperformed competitive methods for other tasks in the field of genetics and genomics, such as predictions of functional effects of genomic sequences [39, 40] and clustering of functional genomics data [41, 42]. The limited accuracy improvement of deep learning-based methods could be attributed to the relative simplicity of the task of learning LD structure. Notably, considering the successful application of the Transformer, it can be safely assumed that adopting more sophisticated deep learning techniques in the future can enable overcoming this limitation [25, 38]. Furthermore, deep learning models can further enhance their performance with the increasing volume of data increases, because which they can significantly outperform other methods for new larger panels [43].

Another potential obstacle for users is that the current deep learning-based genotype imputation methods lack a function to output the imputation reliability metrics like the INFO score implemented in the standard software tools, which enables the filtering out of poorly imputed variants. However, metrics that do not require true genotypes can be calculated for imputation results obtained from deep learning-based methods [44]. Additionally, performing cross-validation using reference panels could be a simple alternative approach for obtaining such filtering criteria [34], although this process can be time-consuming. Furthermore, Bayesian deep learning methods, which allow models to estimate concurrently prediction uncertainties [45], may provide the potential to identify incorrectly imputed variants [34]. Thus, future research should focus on to determining the most suitable reliability measure for deep learning-based imputation through comprehensive benchmarking.

**Fig. 2:**
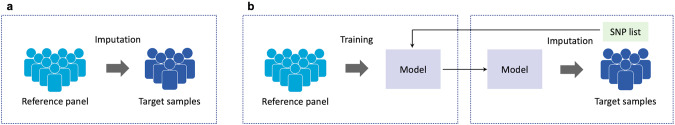
Remote imputation with privacy protection using deep learning-based methods. **a** The standard genotype imputation tools need to be at the same institutions or servers, since they require reference panels with target genotype data to impute. **b** Models can be trained solely with reference panels and SNP lists of target genotype data, and these trained models can be used for performing imputation. Therefore, imputation can be conducted by transferring the SNP lists and trained models, even when reference panels and target genotype data are at different institutions

An inherent advantage of model-based imputation methods is that reference panels are no longer required after models are trained with these panels unlike the standard software tools (Fig. 2a, b). This is a common advantage of imputation methods that construct portable predictive models, such as HIBAG [46] and ADDIT [47], as well as deep learning-based ones. This attribute has been also referred as “reference-free” in some literature [17–19]. Trained models can be publicly distributed or transferred without requiring ethical permission, considering the fundamental impossibility of reconstructing individual genotype information from model parameters [20]. This could further facilitate collaboration between an institution with target genotype data and another institution with reference panels. Furthermore, this advantage can also be harnessed into remote imputation systems. The existing public imputation servers are associated with ethical issues in handling private information, since the users need to upload target individual genotype data (Fig. 2a). In contrast, users would merely need to submit the SNP lists of target genotype data, enabling the creation of models tailored to them using reference panels on the server (Fig. 2b). Subsequently, users could perform imputation locally upon receiving the trained models. Moreover, Dias et al. reported that though not tuned for SNP lists of target genotypes, their autoencoder-based method achieved competitive performance with the standard tools [19]. This highlights the potential of deep learning based-methods to enable us to perform accurate imputation merely by publishing pre-trained models, irrespective of SNP lists (i.e., SNP array platforms) of the target genotype data. Last but not least, the relatively low computational burden, both in terms of processing time and memory usage [25, 34], may further bolster the appeal of deep learning based-methods in the future.

## Conclusion

We have reviewed the existing tools and discussed the challenges and future perspectives associated with deep learning-based genotype imputation methods. Though the current deep learning-based imputation methods may not consistently demonstrate a remarkable improvement over standard tools in terms of prediction accuracy, they possess unique characteristics that could be harnessed for various applications. Specifically, inter-institution or remote imputation with complete privacy protection can be facilitated considering their reference-free characteristics after training. Furthermore, considering that the basic technologies are still evolving, further improvements in deep learning-based-genotype imputation methods can be expected in the future.

## References

[1] Uffelmann E, Huang QQ, Munung NS, de Vries J, Okada Y, Martin AR, et al. Genome-wide association studies. Nat Rev Methods Prim. 2021;1:59 10.1038/s43586-021-00056-9.

[2] Sherry ST. dbSNP: the NCBI database of genetic variation. Nucleic Acids Res. 2001;29:308–11. 10.1093/nar/29.1.308. 11125122 10.1093/nar/29.1.308 PMC29783

[3] Schaid DJ, Chen W, Larson NB. From genome-wide associations to candidate causal variants by statistical fine-mapping. Nat Rev Genet. 2018;19:491–504. 10.1038/s41576-018-0016-z. 29844615 10.1038/s41576-018-0016-z PMC6050137

[4] Wang QS, Huang H. Methods for statistical fine-mapping and their applications to auto-immune diseases. Semin Immunopathol. 2022;44:101–13. 10.1007/s00281-021-00902-8. 35041074 10.1007/s00281-021-00902-8 PMC8837575

[5] Das S, Abecasis GR, Browning BL. Genotype imputation from large reference panels. Annu Rev Genom Hum Genet. 2018;19:73–96. 10.1146/annurev-genom-083117-021602. 10.1146/annurev-genom-083117-021602 29799802

[6] Naj AC. Genotype imputation in genome-wide association studies. Curr Protoc Hum Genet. 2019;102:1–15. 10.1002/cphg.84. 10.1002/cphg.84 31216114

[7] LeCun Y, Bengio Y, Hinton G. Deep learning. Nature. 2015;521:436–44. 10.1038/nature14539. 26017442 10.1038/nature14539

[8] Rubinacci S, Delaneau O, Marchini J. Genotype imputation using the Positional Burrows Wheeler Transform. PLOS Genet. 2020;16:e1009049 10.1371/journal.pgen.1009049. 33196638 10.1371/journal.pgen.1009049 PMC7704051

[9] Das S, Forer L, Schönherr S, Sidore C, Locke AE, Kwong A, et al. Next-generation genotype imputation service and methods. Nat Genet. 2016;48:1284–7. 10.1038/ng.3656. 27571263 10.1038/ng.3656 PMC5157836

[10] Browning BL, Zhou Y, Browning SR. A one-penny imputed genome from next-generation reference panels. Am J Hum Genet. 2018;103:338–48. 10.1016/j.ajhg.2018.07.015. 30100085 10.1016/j.ajhg.2018.07.015 PMC6128308

[11] Li N, Stephens M. Modeling linkage disequilibrium and identifying recombination hotspots using single-nucleotide polymorphism data. Genetics. 2003;165:2213–33. 10.1093/genetics/165.4.2213 PMC1462870 14704198

[12] De Marino A, Mahmoud AA, Bose M, Bircan KO, Terpolovsky A, Bamunusinghe V, et al. A comparative analysis of current phasing and imputation software. PLoS One. 2022;17:1–22. 10.1371/journal.pone.0260177. 10.1371/journal.pone.0260177 PMC9581364 36260643

[13] Consortium IH 3. Integrating common and rare genetic variation in diverse human populations. Nature. 2010;467:52–58. 10.1038/nature09298. 20811451 10.1038/nature09298 PMC3173859

[14] Genomes Project C, Auton A, Brooks LD, Durbin RM, Garrison EP, Kang HM, et al. A global reference for human genetic variation. Nature. 2015;526:68–74. 10.1038/nature15393. 26432245 10.1038/nature15393 PMC4750478

[15] A reference panel of 64,976 haplotypes for genotype imputation. Nat Genet. 2016;48:1279-83. 10.1038/ng.3643. 10.1038/ng.3643 PMC5388176 27548312

[16] Durbin R. Efficient haplotype matching and storage using the positional Burrows–Wheeler transform (PBWT). Bioinformatics. 2014;30:1266–72. 10.1093/bioinformatics/btu014. 24413527 10.1093/bioinformatics/btu014 PMC3998136

[17] Chen J, Shi X. Sparse convolutional denoising autoencoders for genotype imputation. Genes. 2019;10:1–16. 10.3390/genes10090652. 10.3390/genes10090652 PMC6769581 31466333

[18] Song M, Greenbaum J, Luttrell J, Zhou W, Wu C, Luo Z, et al. An autoencoder-based deep learning method for genotype imputation. Front Artif Intell. 2022;5, 10.3389/frai.2022.1028978 10.3389/frai.2022.1028978 PMC9671213 36406474

[19] Dias R, Evans D, Chen SF, Chen KY, Loguercio S, Chan L, et al. Rapid, Reference-Free human genotype imputation with denoising autoencoders. Elife. 2022;11:1–20. 10.7554/elife.75600. 10.7554/eLife.75600 PMC9555874 36148981

[20] Kojima K, Tadaka S, Katsuoka F, Tamiya G, Yamamoto M, Kinoshita K. A genotype imputation method for de-identified haplotype reference information by using recurrent neural network. PLOS Comput Biol. 2020;16:e1008207 10.1371/journal.pcbi.1008207. 33001993 10.1371/journal.pcbi.1008207 PMC7529210

[21] Hochreiter S, Schmidhuber J. Long short-term memory. Neural Comput. 1997;9:1735–80. 10.1162/neco.1997.9.8.1735. 9377276 10.1162/neco.1997.9.8.1735

[22] Cho K, van Merrienboer B, Gulcehre C, Bahdanau D, Bougares F, Schwenk H, et al. Learning phrase representations using RNN encoder–decoder for statistical machine translation. In: Proceedings of the 2014 Conference on Empirical Methods in Natural Language Processing (EMNLP). Stroudsburg, PA, USA: Association for Computational Linguistics; 2014, pp 1724–34.

[23] Guo M-H, Xu T-X, Liu J-J, Liu Z-N, Jiang P-T, Mu T-J, et al. Attention mechanisms in computer vision: a survey. Comput Vis Media. 2022;8:331–68. 10.1007/s41095-022-0271-y.

[24] Vaswani A, Shazeer N, Parmar N, Uszkoreit J, Jones L, Gomez AN, et al. Attention is all you need. IEEE Ind Appl Mag. 2017;8:8–15. 10.1109/2943.974352.

[25] Mowlaei ME, Li C, Chen J, Jamialahmadi B, Kumar S, Rebbeck TR, et al. Split-transformer impute (STI): genotype imputation using a transformer-based model. bioRxiv. 2023, https://www.biorxiv.org/content/10.1101/2023.03.05.531190v1.

[26] Horton R, Wilming L, Rand V, Lovering RC, Bruford EA, Khodiyar VK, et al. Gene map of the extended human MHC. Nat Rev Genet. 2004;5:889–99. 10.1038/nrg1489 15573121

[27] Shiina T, Hosomichi K, Inoko H, Kulski JK. The HLA genomic loci map: expression, interaction, diversity and disease. J Hum Genet. 2009;54:15–39. 10.1038/jhg.2008.5. 10.1038/jhg.2008.5 19158813

[28] MacArthur J, Bowler E, Cerezo M, Gil L, Hall P, Hastings E, et al. The new NHGRI- EBI catalog of published genome-wide association studies (GWAS Catalog). Nucleic Acids Res. 2017;45:D896–D901. 27899670 10.1093/nar/gkw1133 PMC5210590

[29] Débora YCB, Vitor RCA, Bitarello BD, Kelly N, Jérôme G, Diogo M. Mapping bias overestimates reference allele frequencies at the HLA genes in the 1000 Genomes Project Phase I Data. G3 Genes|Genomes|Genetics. 2015;5:931–41. 25787242 10.1534/g3.114.015784 PMC4426377

[30] Dilthey AT, Moutsianas L, Leslie S, McVean G. HLA*IMP-an integrated framework for imputing classical HLA alleles from SNP genotypes. Bioinformatics. 2011;27:968–72. 10.1093/bioinformatics/btr061. 21300701 10.1093/bioinformatics/btr061 PMC3065693

[31] Jia X, Han B, Onengut-Gumuscu S, Chen WM, Concannon PJ, Rich SS, et al. Imputing amino acid polymorphisms in human leukocyte antigens. PLoS One. 2013;8:e64683 10.1371/journal.pone.0064683. 23762245 10.1371/journal.pone.0064683 PMC3675122

[32] Naito T, Okada Y. HLA imputation and its application to genetic and molecular fine-mapping of the MHC region in autoimmune diseases. Semin Immunopathol. 2022;44:15–28. 10.1007/s00281-021-00901-9. 34786601 10.1007/s00281-021-00901-9 PMC8837514

[33] Karnes JH, Shaffer CM, Bastarache L, Gaudieri S, Glazer AM, Steiner HE, et al. Comparison of HLA allelic imputation programs. PLoS One. 2017;12:1–12. 10.1371/journal.pone.0172444. 10.1371/journal.pone.0172444 PMC5312875 28207879

[34] Naito T, Suzuki K, Hirata J, Kamatani Y, Matsuda K, Toda T, et al. A deep learning method for HLA imputation and trans-ethnic MHC fine-mapping of type 1 diabetes. Nat Commun. 2021;12:1639 10.1038/s41467-021-21975-x. 33712626 10.1038/s41467-021-21975-x PMC7955122

[35] Gu J, Wang Z, Kuen J, Ma L, Shahroudy A, Shuai B, et al. Recent advances in convolutional neural networks. Pattern Recognit. 2018;77:354–77. 10.1016/j.patcog.2017.10.013.

[36] Naito T, Satake W, Ogawa K, Suzuki K, Hirata J, Foo JN, et al. Trans‐ethnic fine‐mapping of the major histocompatibility complex region linked to Parkinson’s disease. Mov Disord. 2021;36:1805–14. 10.1002/mds.28583. 33973677 10.1002/mds.28583 PMC8453830

[37] Akiyama Y, Sonehara K, Maeda D, Katoh H, Naito T, Yamamoto K, et al. Genome-wide association study identifies risk loci within the major histocompatibility complex region for Hunner-type interstitial cystitis. Cell Rep Med. 2023;4:101114 10.1016/j.xcrm.2023.101114. 37467720 10.1016/j.xcrm.2023.101114 PMC10394254

[38] Tanaka K, Kato K, Nonaka N, Seita J. Efficient HLA imputation from sequential SNPs data by Transformer. arXiv. 2022. 10.48550/arXiv.2211.06430. 10.1038/s10038-024-01278-x PMC11422163 39095607

[39] Zhou J, Theesfeld CL, Yao K, Chen KM, Wong AK, Troyanskaya OG. Deep learning sequence-based ab initio prediction of variant effects on expression and disease risk. Nat Genet. 2018;50:1171–9. 10.1038/s41588-018-0160-6. 30013180 10.1038/s41588-018-0160-6 PMC6094955

[40] Avsec Ž, Agarwal V, Visentin D, Ledsam JR, Grabska-Barwinska A, Taylor KR, et al. Effective gene expression prediction from sequence by integrating long-range interactions. Nat Methods. 2021;18:1196–203. 10.1038/s41592-021-01252-x. 34608324 10.1038/s41592-021-01252-x PMC8490152

[41] Yuan H, Kelley DR. scBasset: sequence-based modeling of single-cell ATAC-seq using convolutional neural networks. Nat Methods. 2022;19:1088–96. 10.1038/s41592-022-01562-8. 35941239 10.1038/s41592-022-01562-8

[42] Theodoris CV, Xiao L, Chopra A, Chaffin MD, Al Sayed ZR, Hill MC, et al. Transfer learning enables predictions in network biology. Nature. 2023;618:616–24. 10.1038/s41586-023-06139-9. 37258680 10.1038/s41586-023-06139-9 PMC10949956

[43] Taliun D, Harris DN, Kessler MD, Carlson J, Szpiech ZA, Torres R, et al. Sequencing of 53,831 diverse genomes from the NHLBI TOPMed Program. Nature. 2021;590:290–9. 10.1038/s41586-021-03205-y. 33568819 10.1038/s41586-021-03205-y PMC7875770

[44] Ramnarine S, Zhang J, Chen LS, Culverhouse R, Duan W, Hancock DB, et al. When does choice of accuracy measure alter imputation accuracy assessments? PLoS One. 2015;10:1–18. 10.1371/journal.pone.0137601. 10.1371/journal.pone.0137601 PMC4601794 26458263

[45] Kendall A, Gal Y. What uncertainties do we need in Bayesian deep learning for computer vision? Adv Neural Inf Process Syst. 2017, pp. 5575–5585. 10.5555/3295222.3295309.

[46] Zheng X, Shen J, Cox C, Wakefield JC, Ehm MG, Nelson MR, et al. HIBAG - HLA genotype imputation with attribute bagging. Pharmacogenomics J. 2014;14:192–200. 10.1038/tpj.2013.18. 23712092 10.1038/tpj.2013.18 PMC3772955

[47] Choudhury O, Chakrabarty A, Emrich SJ. Highly accurate and efficient data-driven methods for genotype imputation. IEEE/ACM Trans Comput Biol Bioinforma. 2019;16:1107–16. 10.1109/TCBB.2017.2708701. 10.1109/TCBB.2017.2708701 28574365

